# Perfect Spin-filtering in graphene monolayer-bilayer superlattice with zigzag boundaries

**DOI:** 10.1038/srep25361

**Published:** 2016-05-03

**Authors:** Hang Yu, Jun-Feng Liu

**Affiliations:** 1 Department of Physics, South University of Science and Technology of China, Shenzhen, 518055, China

## Abstract

We show that the spontaneous magnetization is formed at the zigzag boundary between monolayer and bilayer graphene by the self-consistent calculation based on Hubbard model. In a monolayer- bilayer graphene superlattice with zigzag boundaries, it is surprising that nearly 100% spin polarization is achieved in the energy window around the Dirac point, no matter the magnetization configuration at two boundaries is parallel or antiparallel. The reason is that the low-energy transport is only influenced by the magnetization at one edge, but not by that at the other. The underlying physics is unveiled by the spin-split band structure and the distribution of the wave-function pertaining to the lowest (highest) subband of electron (hole).

Graphene has been extensively investigated as a promising candidate for spintronics applications due to its small intrinsic spin-orbit^1^,^2^,^3^ and hyperfine interactions^4^. Spintronics aims to inject, manipulate, and detect spins in electronic devices. The generation of a spin-polarized current or a pure spin current is a fundamental prerequisite for construction of spintronic devices. Much experimental effort has been invested in spin injection from ferromagnetic metals to graphene^5^,^6^,^7^. But a high injection efficiency is difficult to reach due to the impedance mismatch between the two materials. At the other hand, two methods have been theoretically suggested to achieve a high spin injection efficiency from graphene to graphene. One is utilizing the induced Rashba spin-orbit coupling in graphene on Ni(111)^8^,^9^. The generation of highly spin-polarized current^10^,^11^,^12^ and pure spin current^13^ has been predicted, but not observed in experiments yet. The other method is utilizing the spontaneous magnetization at zigzag graphene edges. Two-terminal^14^ and three-terminal^15^ spin filters based on graphene with various geometric structure have been proposed. Recent experimental development in the fabrication of perfect zigzag graphene edges^16^ promotes the realization of such devices. And the observations of the spin-valve effect in Co/graphene/Co junctions^17^, as well as the modulated magnetoresistance by vacancy-induced magnetic moment^18^ greatly revive the field of graphene-based spintronics. Besides, the transport and the interface Landau levels in monolayer-bilayer graphene junctions have also attracted much attention^19^,^20^,^21^,^22^,^23^,^24^. However, the magnetic property of zigzag boundary at monolayer-bilayer graphene interface and its application to spintronic devices have not been touched.

In this work, we theoretically investigate the magnetic property of zigzag boundary between monolayer and bilayer graphene and its influence on spin-dependent electronic transport. For convenience, henceforth we refer to such a boundary as zigzag monolayer-bilayer graphene boundary (ZMBGB). By means of the self-consistent calculation based on Hubbard model, it is verified that the spontaneous magnetization is formed at ZMBGB. Furthermore, we propose a monolayer-bilayer graphene superlattice (MBGS) to achieve a really high spin polarization. The result is surprising in that nearly 100% spin polarization is achieved in an energy window around the Dirac point for both parallel and antiparallel magnetizations at two ZMBGBs in a period. It is found that the low-energy transport is only influenced by the magnetization at one ZMBGB, but not by that at the other. The underlying physics is unveiled by the spin-dependent gap in the band structure and the distribution of the wave-function of related subbands. The insensitivity to the magnetic ground-state makes the perfect polarization robust. The range of the energy window can reach 100 meV, which means the large polarization can maintain at room temperature. The sensibility of the window to the electron-phonon interaction^25^,^26^ has been ignored in this work and is worth further studies.

## Model and Methods

**Figure 1:**
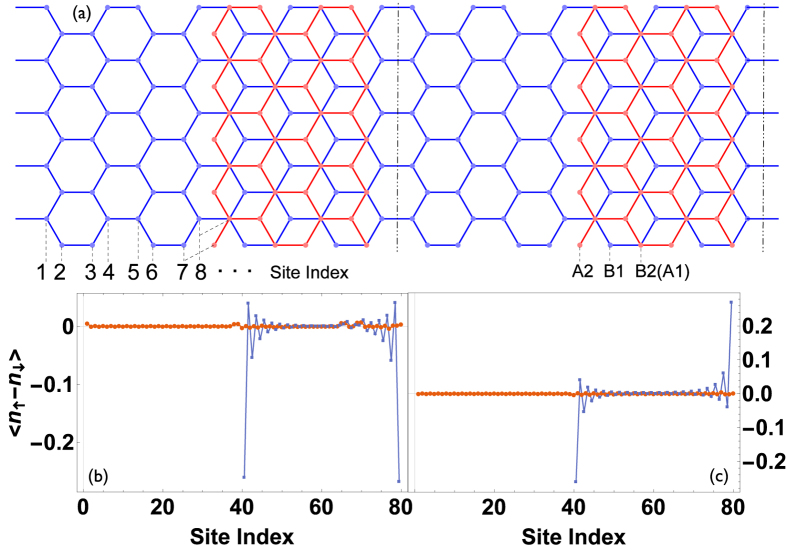
Schematic of the proposed MBGS and its edge magnetizations. (**a**) Is a schematic of the proposed MBGS with zigzag boundaries. The region between two dashed lines represents a period which consists of a monolayer subsection and a bilayer subsection. The atoms are numbered by site indices along the longitudinal direction. (**b**,**c**) show the magnetization at each longitudinal site with (**b**) parallel and (**c**) antiparallel configurations. The upper (lower) layer is represented by the blue (brown) line. *L*_*m*_ = *L*_*b*_ = 20.

We consider a MBGS with zigzag boundaries which is depicted in Fig. 1(a). A-B stacking is considered in bilayer regions. Based on tight-binding and mean-filed Hubbard model, the Hamiltonian can be written as


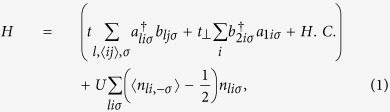


where *a*_*liσ*_ (*b*_*liσ*_) is the annihilation operator at the sublattice A(B), in the layer *l* = 1, 2, at site *i*, and with spin *σ* (=↑ or ↓), and 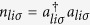 or 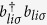 are number operators. *t* = −2.6 eV is the intralayer nearest-neighbor hopping integral, *t*_⊥_ = 0.1*t* is the interlayer nearest-neighbor hopping integral, and *U* = 2.75 eV is the strength of the local on-site Coulomb interaction between opposite spins.

We assume the MBGS lies in the *x*-*y* plane with *y* (*x*) being parallel (perpendicular) to the monolayer-bilayer interface. The regions of monolayer and bilayer graphene have respectively the lengths *L*_*m*_ and *L*_*b*_ which count the numbers of zigzag chains and increase by 1 when the physical lengths increase by 3*a*/2 with *a* the c-c bond length. Thus the length of a period is 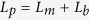. In our work, we have not considered possible reconstructions of ZMBGBs. And we assume that the edge atoms at ZMBGBs are terminated by hydrogen atoms so that the edge magnetization is totally contributed by the edge-localized *π*-orbital state which is well described by the Hubbard model. At first, we consider that system is translational invariant along the *y* direction. For such a MBGS, the Hamiltonian can be reduced to a matrix with finite dimension describing a supercell for given longitudinal and transversal Bloch wave-vectors *k*_*x*_, *k*_*y*_. By numerically diagonalizing the Hamiltonian matrix, we obtain the band structure and corresponding wave-functions for all the Bloch states. The spin-resolved local density of states (DOS) 〈*n*_*liσ*_〉 can be calculated by


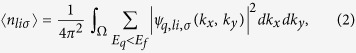


where 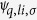 is the *q*-th eigenstate for fixed *k*_*x*_, *k*_*y*_, *E*_*q*_ is the corresponding energy, and *E*_*f*_ = 0 is the Fermi energy. The integral is over the reduced first Brillouin zone Ω. Equations (1) and (2) are solved self-consistently for obtaining the converged 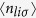. As shown in the Hamiltonian, the difference of local DOS between opposite spins 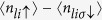 acts as an effective exchange potential, which is responsible for the spin-dependent transport.

When the spin-dependent transport is considered, the *x*-axis is taken as the transport direction. For the purpose of practical applications, we consider that the MBGS has finite width *N* along the *y* direction. *N* is an integer which increases by 1 when the physical width increases by 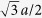.

To study the transmission and conductance, we cut a few periods of monolayer-bilayer junctions out of the superlattice and sandwich them between two monolayer graphene ribbon leads. The spin dependent ballistic conductance is calculated using the Landauer-Büttiker formalism


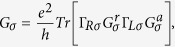


where 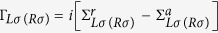 describes the coupling of the device to left (right) lead. The Green functions read


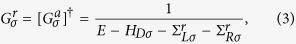


where *H*_*Dσ*_ is the Hamiltonian of the device region, and the retarded self-energy 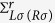 due to the coupling to the terminal L(R) can be calculated numerically. The total conductance and the polarization are defined respectively as


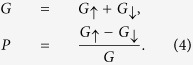


## Results and Discussions

Figure 1(b,c) show the edge magnetizations formed at the two edges of the upper layer (layer 2). It is similar to in the monolayer graphene zigzag ribbon that both parallel and antiparallel configurations are possible because the difference of ground-state energy becomes ignorable when 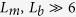. The magnetizations are mainly located at the outmost sites at two sides of layer 2. The value is approximately 0.27*t*, slightly weaker than that in monolayer graphene zigzag ribbon. Without loss of generality, we assume that the leftmost site A2 is not directly coupled to layer 1 while the rightmost site B2 is directly coupled to A1 (see Fig. 1(a)). It is shown that the left magnetization is slightly weaker than the right one in both parallel and antiparallel cases.

**Figure 2:**
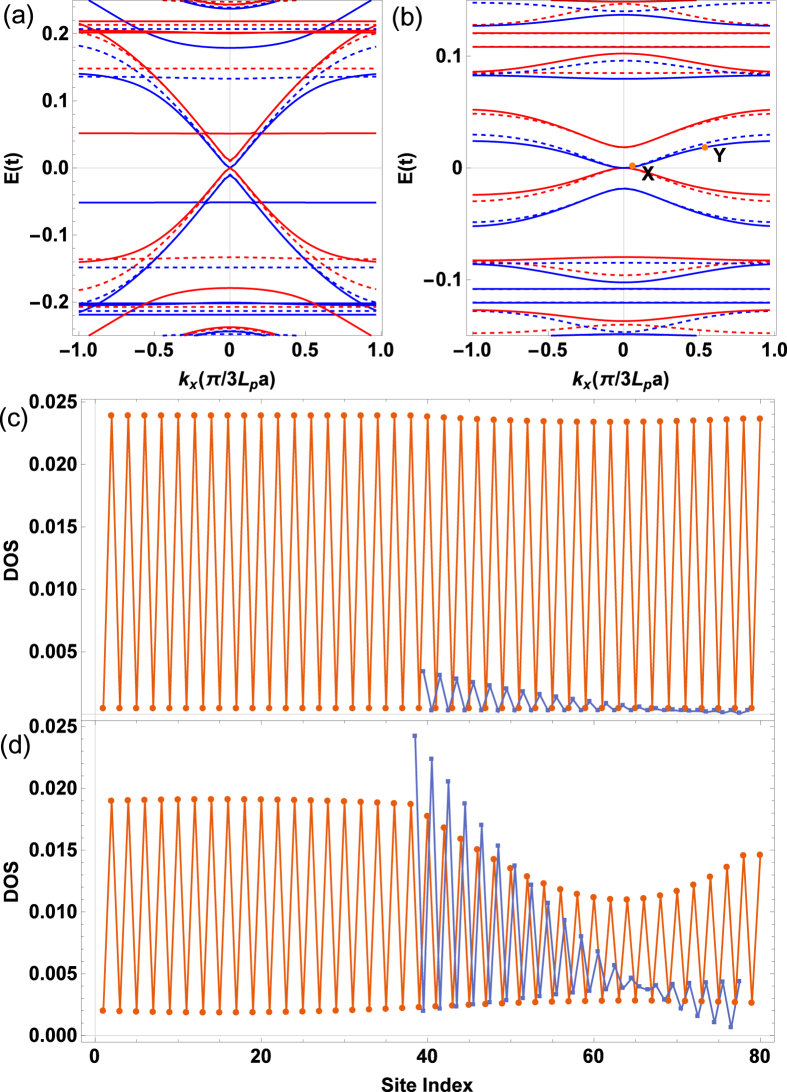
Spin-split band structures and the wave function distribution for two states in the lowest electronic band. (**a**,**b**) Are spin-split band structure for MBGS with finite width *N* = 20 in both antiparallel (solid lines) and parallel (dashed lines) configurations. The blue (red) curves stand for spin-up (spin-down). The other parameters are *L*_*m*_ = *L*_*b*_ = 6 for (**a**), and *L*_*m*_ = *L*_*b*_ = 20 for (**b**). (**c**,**d**) Are local DOS at each longitudinal site for two marked states X (**c**) and Y (**d**) in (**b**) for spin-up electrons in the antiparallel configuration. The upper (lower) layer is represented by the blue (brown) line. The local DOS have been summed up over all transverse sites.

Since the edge magnetizations are confirmed at ZMBGBs in MBGS, we now discuss the consequence in the spin-polarized transport. For practical device applications, we consider a MBGS with finite transversal width *N* = 20. The band structures for various parameters are shown in Fig. 2(a,b). The magnetizations result in the spin-splitting of the band structure. For each spin, the magnetizations also open a gap in the band structure near the Dirac point. We denote the spin-splitting energy as Δ, which is also the gap for each single spin. In the energy region [0, Δ], only spin-up band is open. While for [−Δ, 0], only spin-down band is open. Therefore, a perfect spin-filtering effect can be supported in the energy region [−Δ, Δ]. It is interesting that no matter the magnetization configuration is antiparallel or parallel, the band structure is nearly the same in the low-energy regime. This important property makes the perfect spin-filtering effect robust and is very important to the practical applications. Note that the two valleys have been mixed by the transversal scattering along zigzag directions and no valley-filtering effect is accompanied as in MoS_2_^27^.

The underlying physics can be unveiled by exploring the wave-function distribution, or the local DOS of the low-energy states. As in the monolayer graphene zigzag ribbon, the DOS at A2 sites decrease from left to right while the DOS at B2 sites decrease from right to left (see Fig. 2(c,d)). It is more important that the wave-function for a propagating state in the bilayer graphene is mainly distributed to weakly coupled nearest sites A2 and B1^28^. The distribution to strongly coupled sites B2 and A1 is very little. As a result, the DOS is very small in the right side of layer 2. It is shown in Fig. 1(b,c) that the right magnetization is mainly located at the outmost B2 sites. The small distribution of wave-function at B2 sites means that the right magnetization has ignorable influence on the low-energy transport.

**Figure 3:**
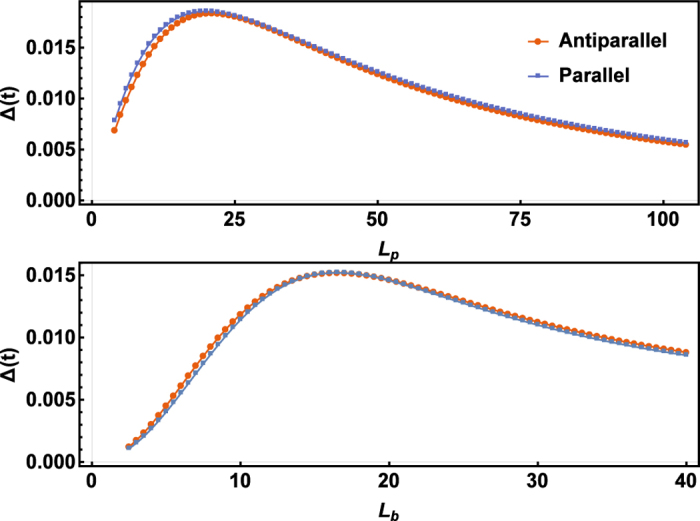
The dependence of the spin-splitting energy on structure parameters. Spin-splitting energy at *k*_*x*_ = 0 as the function of structure parameters *L*_*p*_ (**a**) and *L*_*b*_ (**b**) for MBGS with width *N* = 20 in both antiparallel and parallel configurations. The ratio *L*_*m*_/*L*_*b*_ = 1 is fixed in (**a**) and *L*_*p*_ = 90 is fixed in (**b**).

Figure 3 shows the dependence of spin-splitting energy Δ on structure parameters *L*_*p*_ and *L*_*b*_. The lengths of monolayer and bilayer regions *L*_*m*_, *L*_*b*_ can be optimized to obtain the maximal Δ. The result is nearly the same for antiparallel and parallel configurations. The maximum of Δ can reach nearly 50 meV, which implies the feasibility of experimental observation of perfect spin-filtering effect at room temperature.

**Figure 4:**
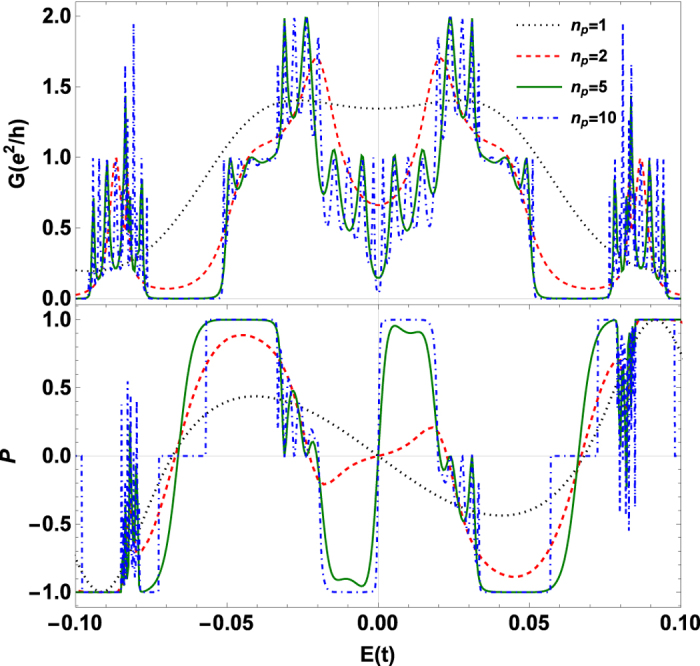
Conductance and polarization. Conductance (**a**) and Polarization (**b**) as functions of the Fermi energy for various numbers of periods *n*_*p*_ out of the MBGS in the device region. The antiparallel configuration is considered. The other parameters are the same as those in Fig. 2(b).

In the practical applications, the device region is finite. We cut several periods of monolayer-bilayer junctions out of the superlattice and sandwich them between two monolayer graphene ribbon leads. Figure 4(a,b) show the dependence of conductance and polarization on the number of periods *n*_*p*_. The conductance exhibits much oscillation due to the multi-reflection by the leads. With increasing *n*_*p*_, the oscillation becomes heavier because of the larger length of device region. For perfect spin polarization, we focus on the energy window [−Δ, Δ]. When *n*_*p*_ = 1, the polarization is opposite to that for *n*_*p*_ ≥ 2 in the energy window. With increasing *n*_*p*_ from 2, the polarization increases. When *n*_*p*_ increases up to 10, the polarization becomes nearly 100%. That is because the device region is approximately a superlattice and well described by the spin-split band structure shown in Fig. 2(b). It means that the approximate superlattice structure is crucial to achieve a really high spin polarization.

## Conclusion

In summary, we propose a perfect spin-filter based on a MBGS and predict a nearly 100% spin polarization in the energy window [−50, 50] meV around the Dirac point. The edge magnetizations at zigzag boundaries of MBGS and the superlattice structure are responsible for the perfect spin polarization. It is remarkable that the perfect polarization can survive no matter the magnetic ground-state is antiparallel or parallel. It means that the proposed perfect spin-filter is very robust and can work at room temperature.

## Additional Information

**How to cite this article**: Yu, H. and Liu, J.-F. Perfect Spin-filtering in graphene monolayer-bilayer superlattice with zigzag boundaries. *Sci. Rep.* **6**, 25361; doi: 10.1038/srep25361 (2016).
